# Inhibition of cytokine gene expression and induction of chemokine genes in non-lymphatic cells infected with SARS coronavirus

**DOI:** 10.1186/1743-422X-3-17

**Published:** 2006-03-29

**Authors:** Martin Spiegel, Friedemann Weber

**Affiliations:** 1 Abteilung Virologie, Institut für Medizinische Mikrobiologie und Hygiene, Universität, Freiburg, D-79008 Freiburg, Germany

## Abstract

**Background:**

SARS coronavirus (SARS-CoV) is the etiologic agent of the severe acute respiratory syndrome. SARS-CoV mainly infects tissues of non-lymphatic origin, and the cytokine profile of those cells can determine the course of disease. Here, we investigated the cytokine response of two human non-lymphatic cell lines, Caco-2 and HEK 293, which are fully permissive for SARS-CoV.

**Results:**

A comparison with established cytokine-inducing viruses revealed that SARS-CoV only weakly triggered a cytokine response. In particular, SARS-CoV did not activate significant transcription of the interferons IFN-α, IFN-β, IFN-λ1, IFN-λ2/3, as well as of the interferon-induced antiviral genes ISG56 and MxA, the chemokine RANTES and the interleukine IL-6. Interestingly, however, SARS-CoV strongly induced the chemokines IP-10 and IL-8 in the colon carcinoma cell line Caco-2, but not in the embryonic kidney cell line 293.

**Conclusion:**

Our data indicate that SARS-CoV suppresses the antiviral cytokine system of non-immune cells to a large extent, thus buying time for dissemination in the host. However, synthesis of IP-10 and IL-8, which are established markers for acute-stage SARS, escapes the virus-induced silencing at least in some cell types. Therefore, the progressive infiltration of immune cells into the infected lungs observed in SARS patients could be due to the production of these chemokines by the infected tissue cells.

## Background

For most viruses, the initial encounter with the host takes place in cells of non-lymphatic origin. The outcome of this primary infection can determine the course of disease, and the cytokine response of the infected cell plays a vital part. Type I interferons (IFN-α/β) are potent, antivirally active cytokines which can be produced by most, if not all, body cells in response to virus infection. IFNs not only trigger the synthesis of antivirally active proteins, they also activate the innate immune system and help to shape adaptive immunity [1]. Other virus-induced cytokines and chemokines activate the adaptive immune system and direct the migration of leukocytes [2]. Viruses, on the other hand, have evolved various mechanisms to counteract the host's cytokine response [3], and their ability to induce or inhibit cytokine production in infected cells has direct consequences for the balance between host defense and virus propagation.

SARS coronavirus (SARS-CoV) is the etiologic agent of severe acute respiratory syndrome (SARS), a life-threatening new human disease which recently emerged in China [4-7]. Characteristic SARS symptoms are high fever, myalgia, dry cough and lymphopenia, and in around 30% of cases patients also developed an atypical form of pneumonia [8].

The mechanisms underlying SARS-CoV-mediated pathogenesis remain largely unexplained. Autopsies from deceased patients revealed severe damage of the lungs and lymphatic tissues, accompanied by infiltrations of monocytic cells [9-11]. This may indicate that immunopathogenesis is involved in the severe outcome of the disease, providing the rationale for SARS therapy with immunosuppressant corticosteroids [12]. On the other hand, there is evidence that cell damages could be directly caused by the virus, since SARS-CoV is cytolytic [13] and capable to systemically infect human hosts [14-16]. In addition, virus particles and signs of necrosis were found in affected tissues [11], and high viral loads are predictive of adverse clinical outcome [17]. Interestingly, however, the acute lung injuries and respiratory failure observed in severe cases occured while viral loads were declining [16], again favouring the hypothesis of immune-mediated lung damage.

Virus-induced cytokines not only play a significant role in host defense, but also in immunopathogenesis. Investigations of the cytokine profiles of SARS patients have shown that the proinflammatory cytokines and chemokines IL-6, IL-8 and IP-10 (CXCL10) are strongly upregulated [18-23]. Cell culture studies, by contrast, did not reveal a clear picture of SARS-CoV-induced cytokines. In some cases both IL-8 and IP-10 were upregulated [24], whereas in other cases either only IL-8 [25,26], only IP-10 [27], or no cytokines were induced at all [28]. IL-6 was only moderately upregulated [29], or not detected at all [24-26]. Thus, it is still unclear whether the cytokine storm in SARS patients was directly caused by the virus, i.e. produced by SARS-CoV-infected cells, or whether it is a secondary effect, i.e. the result of strong activation of the immune system.

With one notable exception [24], most studies investigating the cellular cytokine response to SARS-CoV were either based on immune cells [25,27-29] or on Huh7 hepatoma cells inoculated with unphysiologically high amounts of virus [26]. Thus, the overall picture of the cytokine response of non-immune cells, which are most probably the prime targets of SARS-CoV, may still be incomplete. To learn more about it, a human cell line would be needed which, on one hand, can support the complete viral replication cycle, but on the other hand is also able to produce cytokines which are potentially antiviral. However, most cell lines which are permissive for SARS-CoV have lost the ability to synthesize IFNs, the most potent antiviral cytokines [24,30,31]. In this study, we identified an IFN-competent human embryonic kidney (HEK) 293 cell clone which supports the growth of SARS-CoV. Using these cells as well as the established human colon carcinoma cell line Caco-2 [24,31], we investigated the SARS-CoV-induced production of representative cytokines, chemokines and antiviral genes. Our studies revealed that SARS-CoV is capable to suppress the antiviral cytokine response of infected cells to a large extent. Interestingly, however, induction of the chemokines IP-10 and IL-8 escaped suppression by SARS-CoV in Caco-2 cells, but not in HEK 293s. Thus, SARS-CoV efficiently blocks the innate host cell defense at a very early step of infection, buying time to colonize the host. With the possible exception of IP-10 and IL-8, most cytokines detected in SARS patients may therefore be produced by the infiltrating immune cells, and not by the resident tissue cells. These data may help to explain both the rapid rise in virus titers during the initial stage of disease, caused by the suppression of antiviral cytokines, as well as the progressive infiltration of immune cells into the infected lungs, which could be due to the production of chemokines by the infected tissue cells.

## Results

### Growth of SARS-CoV in different cell lines

**Figure 1:**
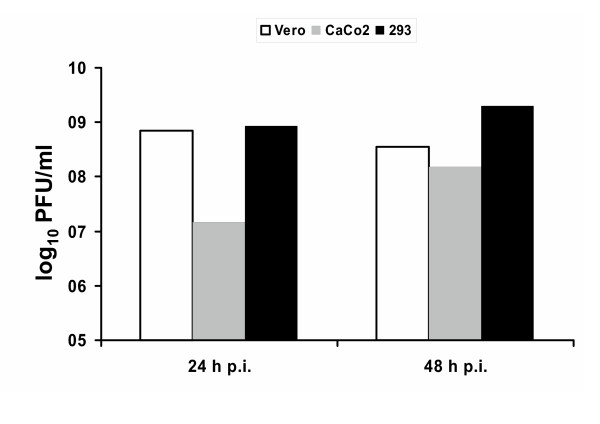
**Virus titers**. Simian Vero cells (white bars), human Caco-2 cells (grey bars), and human low-passage HEK 293 cells (black bars) were infected at a multiplicity of infection (MOI) of 5 infectious particles per cell. Virus titers in the supernatants were determined 24 h post-infection and 48 h post-infection by plaque assays.

Vero cells, which are standard for growth of SARS-CoV [30,31], lack type I IFN genes [32,33] and therefore are not suitable for cytokine analyses. In search of an appropriate *in vitro *system, we tested several IFN-competent human cell lines for SARS-CoV growth and identified a low-passage clone of HEK 293 cells [34] as being fully permissive. Fig. 1 shows that titers of HEK 293 cells and Vero cells were comparable and rather high already at 24 h post-infection. Caco-2 cells, by contrast, produce approximately 100-fold less virus at 24 h post-infection, and 10-fold less at 48 h post-infection (Fig. 1). Thus, we considered both the Caco-2 cells and the low passage HEK 293 cells as useful systems for studying the influence of SARS-CoV on the immune system-independent induction of cytokines.

### Interferon genes and their antiviral effectors

To properly assess the cytokine profile of SARS-CoV infection, we compared it with well-characterized cytokine inducers such as Bunyamwera delNSs virus (BdNSs [35]), Sendai virus (SeV) and Newcastle disease virus (NDV). In addition, we deemed it necessary to monitor cytokine synthesis both at 8 h and at 16 h post-infection, since we previously found a striking difference between the early and the late host cell response to SARS-CoV [36].

**Figure 2:**
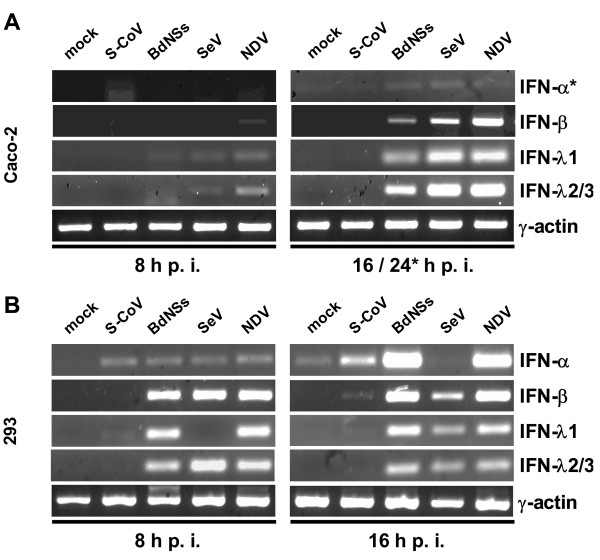
**Interferon production by virus-infected human cells**. Caco-2 cells (A) and HEK 293 cells (B) were infected with SARS-CoV or the IFN-inducing control viruses Bunyamwera delNSs (BdelNSs), Sendai virus (SeV), Newcastle disease virus (NDV), or were left uninfected (mock). At 8 h (left panels) or at 16 h (right panels) post-infection, total RNA was isolated and investigated by RT-PCR for the presence of different IFN mRNAs. The cellular γ-actin mRNA served as loading control. Note that for the reliable detection of IFN-α in Caco-2 cells (A, upper right panel) the infection time had to be extended to 24 h.

**Figure 3:**
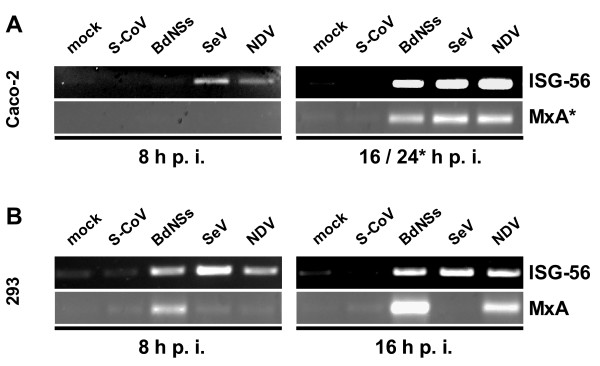
**Interferon-stimulated genes**. RNA samples of Caco-2 cells (A) and HEK 293 cells (B) described in Fig. 2 were investigated by RT-PCR for the presence of ISG56 and MxA mRNAs. As for IFN-α (see Fig. 2A), for detection of MxA mRNA in Caco-2 cells an extended infection period of 24 h was necessary (A, lower right panel).

The first set of tested cytokines comprised the classical antiviral cytokines IFN-α and IFN-β [1], and the novel interferons IFN-λ1 and IFN-λ2/3 [37]. To test their induction in cell culture, we infected with 5 plaque-forming units (pfu) of viruses per cell, and analyzed cytokine mRNAs by RT-PCR. As it is shown in (Fig. 2A and 2B), clear signals for all IFNs were detected after infection with the control viruses BdNSs, SeV and NDV. For SARS-CoV, by contrast, only a weak signal for IFN-α was detected in HEK 293 cells, and none for IFN-β or the IFN-λs in either cell line. All preparations contained similar amounts of input RNA, since the γ-actin control mRNA was present in equal amounts (Fig. 2A and 2B, lower panels). It was of interest to see whether virus infection would lead to the upregulation of antiviral, IFN-stimulated genes (ISGs). As specific and sensitive markers we used the ISG56 gene which is induced both by IFNs and by virus infection [38,39] and the MxA gene which is exclusively activated by IFNs [40]. As is evident from Fig. 3, no significant ISG induction occurs for SARS-CoV, whereas the control viruses activated ISG expression. Note that SeV blocks in HEK 293 cells the synthesis of IFN-α (see Fig. 2B, upper right panel) and of MxA (Fig. 3B, lower right panel), most probably because of its ability to inhibit IFN-induced signaling [41]. Curiously, this does not happen in Caco-2 cells (Fig. 3A, lower right panel), suggesting cell type-specific differences in cytokine signaling.

Taken together, these data demonstrate that, in contrast to the other viruses tested, SARS-CoV suppresses the activation of the antiviral IFNs and the IFN-induced effector genes to a large extent.

### Induction of chemokines by infected cells

**Figure 4:**
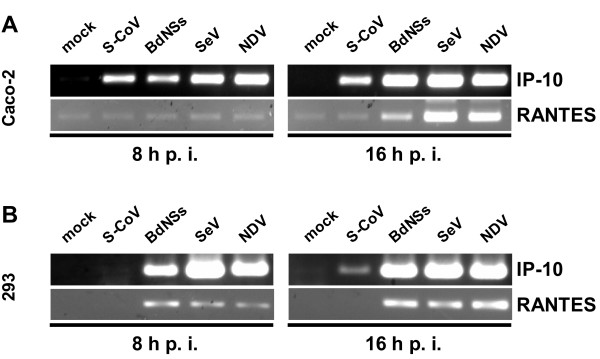
**Chemokine production**. RNA samples of Caco-2 cells (A) and HEK 293 cells (B) described in Fig. 2 were assayed by RT-PCR for IP-10 and RANTES mRNA levels.

IP-10 and RANTES are potent chemoattractants for activated T cells and NK cells [2]. When we infected Caco-2 cells, significant amounts of IP-10 mRNA were synthesized (Fig. 4A, upper panels). This strong upregulation occurred independently of the virus, suggesting a general response to virus infection. Although IP-10 mRNA levels induced at 16 h p.i. by SARS-CoV are slightly lower than by the other viruses, our data are in good agreement with previous studies [24,27]. Induction of RANTES, by contrast, was restricted to the cytokine-inducing viruses, whereas infection with SARS-CoV had no effect above background levels (Fig. 4A, lower panels). We then tested HEK 293 cells in a similar way. Much to our surprise, IP-10 mRNA was not detectable early after infection with SARS-CoV (Fig. 4B, upper left panel), and only very weakly expressed after longer infection (Fig. 4B, upper right panel).

RANTES mRNA again was not detectable for SARS-CoV (Fig. 4B, lower panels). All three cytokine-inducing viruses activated IP-10 and RANTES expression in HEK 293 cells as expected (Fig. 4B, upper and lower panels).

Thus, SARS-CoV induces IP-10 gene expression in Caco-2 cells, but not in HEK 293 cells, again suggesting that the cytokine response is dependent on the host cell type. RANTES expression, by contrast, is never induced by SARS-CoV, although the cells respond normally to other viruses.

### Induction of IL-6 and IL-8

**Figure 5:**
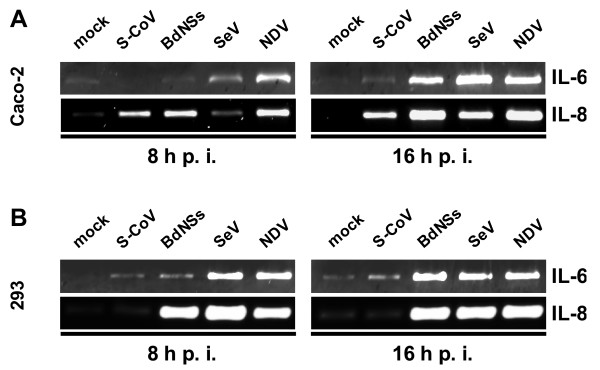
**Interleukin production**. RNA samples of Caco-2 cells (A) and HEK 293 cells (B) described in Fig. 2 were investigated by RT-PCR for the presence of IL-6 and IL-8 mRNAs.

The proinflammatory cytokine IL-6 and the chemokine IL-8 are strongly upregulated in SARS patients [18,19], but from cell culture studies no clear picture emerged [24-26,29]. We investigated IL-6 and IL-8 production by Caco-2 and HEK 293 cells infected with SARS-CoV and compared it with the other RNA viruses. As shown in Fig. 5, IL-6 is induced only weakly by SARS-CoV, independent of the cell line used (Fig. 5A and 5B, upper panels). IL-8, by contrast, is clearly induced by SARS-CoV in Caco-2 cells, but not in HEK 293 cells (Fig. 5A and 5B, lower panels). The control viruses invariably induced both IL-6 and IL-8, demonstrating that the cell lines are capable to produce these cytokines.

Thus, SARS-CoV strongly induces IL-8, but not IL-6 in a cell-type dependent manner. This may suggest that the IL-8 detected in SARS patients [18,19] is directly synthesized by infected resident cells, whereas IL-6 is more likely a secondary response mediated by infiltrating immune cells.

Taken together, our data demonstrate that SARS-CoV in general is a weak inducer of cytokines and antiviral genes in non-lymphatic cells. Chemokines like IP-10 and IL-8, however, can be directly upregulated in SARS-CoV in a cell-type-dependent manner.

## Discussion

The activation of immune-relevant cytokines and host cell genes by SARS-CoV in cells and patients was the subject of several previous investigations [18,21,22,24-29,42-44]. However, most of the cell culture studies were either based on immune cells which do not represent the majority of infected cells [25,27-29], or on Huh7 hepatoma cells which needed to be infected with 100 pfu per cell, i.e. with unphysiologically high amounts of virus [26]. Moreover, Huh7 cells are known to be deficient in the antiviral cytokine response [45]. Thus, it was not entirely clear whether the patients' cytokine response was caused by virus-infected cells, or whether it was mediated by the activated immune system. Furthermore, it was not systematically investigated how the cytokine induction by SARS-CoV compares to other viruses. Here, we have used three control viruses and two different cells lines to elucidate and compare the induction of cytokines by SARS-CoV. Our results demonstrate that SARS-CoV does not induce significant amounts of IFNs, antiviral genes, RANTES, and IL-6. In agreement with this finding, SARS-CoV-infected macrophages and dendritic cells lack IFN induction [27,29]. IP-10 and IL-8, however, can be activated by SARS-CoV. This suggests that these chemokines, which are reliable markers of acute-stage SARS [18,20,21,23], are not only produced in response to IFN-γ after activation of the immune system as suggested, but may also be directly secreted by infected tissue cells. An upregulation of either IP-10 and/or IL-8 was observed in several studies using SARS-CoV-infected Caco-2 cells [24], macrophages [27], peripheral blood mononuclear cells [25], and dendritic cells [29]. Using HEK 293 cells, by contrast, we found that SARS-CoV is able to downregulate also IP-10 and IL-8 production. Similarly, recent studies showed that peripheral blood monocytes from SARS patients do not produce any cytokines [28]. Thus, chemokine induction by SARS-CoV appears to be highly cell type-specific.

With the exception of IP-10 and IL-8, SARS-CoV is capable to suppress the production of a wide range of cytokines. This is in agreement with our previous finding that SARS-CoV inhibits the crucial cytokine transcription factor IRF-3 [36], providing a possible mechanism for the high potential of this pathogen to suppress the host response. Of note, SARS-CoV is highly sensitive to the antiviral action of IFNs both in vivo and in vitro [46-51], thus explaining why the virus needs to suppress IFN induction in advance.

## Conclusion

In the initial phase of SARS, the virus grows exponentially and spreads to different organs, including the lungs [8,14]. Our data may explain this rapid and efficient dissemination of SARS-CoV. By slowing down expression of IFNs and their antiviral genes in the infected tissue cells, the virus buys time during the initial, critical phase of infection in order to grow unhindered in the host. At the same time, however, the virus-induced chemokines IP-10 and IL-8 attract immune cells. Possibly, this mixture of high-level virus replication followed by the invasion of activated immune cells results in a strong inflammatory response, leading to a cytokine storm and the severe and potentially fatal respiratory distress which is the hallmark of full-blown SARS.

## Methods

### Cells and viruses

Simian VeroE6 cells, human Caco-2 cells and human embryonic kidney (HEK) 293 cells were maintained and grown as described [24,36]. The low-passage HEK 293 cell clone [34] was purchased from Microbix Biosystems, Toronto, Canada. All experiments were performed with HEK 293 cells between passage 38 and 48. The FFM-1 isolate of SARS-CoV was kindly provided by Stephan Becker, University of Marburg, Germany. Bunyamwera delNSs virus [35], Sendai virus and Newcastle disease virus were used as controls.

### Plaque assays

Virus plaque assays were performed as described previously [50]. Briefly, Vero cell monolayers were infected with dilutions of supernatants from infected cells, overlaid with soft agar, and allowed to form plaques for 72 h. Then the agar overlay was removed and cells were stained with a solution of 1% crystal violet, 3,6% formaldehyde, 1% methanol, and 20% ethanol.

### RT-PCR analyses

Cells were infected for the indicated times, total RNA was extracted and treated with DNase I. For reverse transcription (RT), 1 μg of RNA was incubated with 200 U of Superscript II reverse transcriptase (Invitrogen) and 100 ng random hexanucleotides in 20 μl of 1×RT buffer (Invitrogen) supplied with 1 mM each of the four deoxynucleotide triphosphates, 20 U of RNasin, and 10 mM dithiothreitol. The resulting cDNA was amplified by 35 cycles of PCR, with each cycle consisting of 30 sec at 94°C, 1 min at 58°C (using primer pairs specific for IP-10, IL-6, IL-8 and RANTES) or at 56°C (all other primer pairs), and 1 min at 72°C, followed by 10 min at 72°C. Primer sequences are available from the authors upon request.

## List of abbreviations

BdNSs, Bunyamwera delNSs virus; HEK, human embryonic kidney; IFN, interferon; IL, interleukine; ISG, interferon-stimulated gene; NDV, Newcastle disease virus; RANTES, Regulated on activation, normal T cell expressed and secreted; SARS-CoV, SARS coronavirus; SeV, Sendai virus

## Competing interests

The author(s) declare that they have no competing interests.

## Authors' contributions

MS carried out the virus growth studies and the RT-PCR analyses, participated in the design of the study, and has given final approval of the version to be published. FW carried out virus infections, participated in the design of the study, and was responsible for drafting and finalizing the manuscript. All authors read and approved the final manuscript.
